# THE EFFECTS OF CONE-BEAM COMPUTED TOMOGRAPHY IMAGING GUIDANCE ON PATIENT RADIATION EXPOSURES IN TRANS-ARTERIAL CHEMOEMBOLISATION FOR HEPATOCELLULAR CARCINOMA

**DOI:** 10.1093/rpd/ncac077

**Published:** 2022-05-30

**Authors:** S Y Wong, S Foley, C P Cantwell, R Ryan, J Lucey, P Maher, J P McNulty

**Affiliations:** Radiography and Diagnostic Imaging, School of Medicine, University College Dublin, Dublin 4, Ireland; Department of Radiology, St. Vincent’s University Hospital, Elm Park, Dublin 4, Ireland; Department of Diagnostic Radiology, Singapore General Hospital, Outram Road, Singapore 169608, Singapore; Radiography and Diagnostic Imaging, School of Medicine, University College Dublin, Dublin 4, Ireland; Department of Radiology, St. Vincent’s University Hospital, Elm Park, Dublin 4, Ireland; Department of Radiology, St. Vincent’s University Hospital, Elm Park, Dublin 4, Ireland; School of Medicine, University College Dublin, Dublin 4, Ireland; Department of Radiology, St. Vincent’s University Hospital, Elm Park, Dublin 4, Ireland; School of Medicine, University College Dublin, Dublin 4, Ireland; Department of Radiology, St. Vincent’s University Hospital, Elm Park, Dublin 4, Ireland; School of Medicine, University College Dublin, Dublin 4, Ireland; Department of Radiology, St. Vincent’s University Hospital, Elm Park, Dublin 4, Ireland; Radiography and Diagnostic Imaging, School of Medicine, University College Dublin, Dublin 4, Ireland; Department of Radiology, St. Vincent’s University Hospital, Elm Park, Dublin 4, Ireland

## Abstract

This study investigated the effects of cone-beam computed tomography (CBCT) guidance in trans-arterial chemoembolisation (TACE) procedures on the number of digital subtraction angiography (DSA) runs acquired and total patient radiation exposure in patients with hepatocellular carcinoma (HCC). A retrospective, analytical cross-sectional, single institution, study was conducted. Dose data were compared across the control (DSA guidance alone) and study (DSA and CBCT guidance) groups. A total of 122 procedures were included within the study. There was a significant reduction in the number of DSA runs (3 vs 5, *p < 0.001*) and DSA air kerma-area product (PKA) (3077.3 vs 4276.6 μGym^2^, *p = 0.042*) for the study group when compared to the control group. Total procedural PKA and total procedural reference air kerma (K_a,r_) were shown to be 50 and 73% higher, respectively, for the study group when compared to the control group. CBCT imaging guidance does reduce the number of DSA runs and DSA PKA required to complete the TACE procedure for patients diagnosed with HCC; however, a substantial increase in total procedural PKA is to be expected and it is thus important that this increased dose is carefully considered and justified.

## INTRODUCTION

The advent of C-arm cone-beam computed tomography (CBCT) within the interventional suite has enabled interventionists to obtain 3D volumetric imaging on the procedure table. This is in addition to the 2D imaging of fluoroscopy, direct radiography, and digital subtraction angiography (DSA) routinely used for image-guided interventions^(1)^. The technology is especially pertinent to hepatocellular carcinoma (HCC) patients undergoing trans-arterial chemoembolisation (TACE) procedures as CBCT has altered the way TACE procedures are being performed today^(2, 3)^. Many studies have demonstrated the advantages of CBCT in TACE procedures^(2–4)^, in particular increased safety, minimising complications and making procedures technically easier to perform.

As exposure dose calculations for fluoroscopy-guided procedures are complex, four different parameters were reviewed to evaluate patient radiation exposure. In addition to the number of DSA runs performed for each procedure, the air kerma-area product (PKA), reference air kerma (K_a,r_) and fluoroscopy times were measured and analysed. PKA reflects the energy of the entire X-ray beam and thus total energy delivered to the patient while the K_a,r_ being the air kerma at a specific point (15 cm from the isocentre) along the central beam and has been recommended as the best approximation of skin dose for consideration of potential deterministic tissue reactions from radiation exposure^(5, 6)^.

This study was performed to investigate the effects of CBCT guidance in TACE procedures on the number of DSA runs acquired and total patient radiation exposure in patients with HCC.

## PATIENTS AND METHODS

This study was performed as a retrospective, cross-sectional, single institution, study and did not require full institutional ethical approval. An ethical exemption was issued by the Institutional Review Board (reference: LS-E-17-59-Wong-McNulty). To remove equipment variability, all procedures were performed on a single angiographic equipment system (Siemens Artis Q ceiling mounted unit; Siemens, Erlangen, Germany) installed in January 2014. All patient data within this study were retrieved from the radiology information system (RIS) and picture archiving and communication system (PACS). Specific dose readings for each procedure were available from the radiation dose structured report (RDSR) stored within PACS for each procedure.

### Protocol

**Figure 1:**
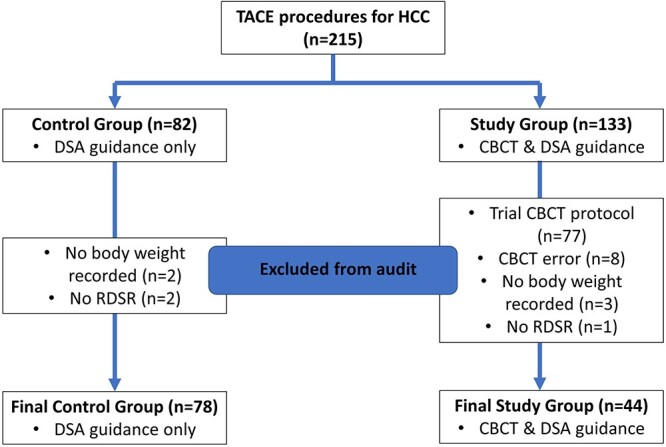
Flow diagram of TACE procedure enrolment and sample number (*n*) for both control and study groups (CBCT = Cone-beam computed tomography; DSA = Digital subtraction angiography; HCC = Hepatocellular carcinoma; RDSR = Radiation dose structured report; TACE = Trans-arterial chemoembolisation).

All consecutive patients who underwent TACE procedures over a period of 3 years and 2 months since installation, and prior to a firmware and hardware upgrade, were considered for inclusion. Inclusion and exclusion criteria are detailed in Table 1. Patients’ diagnoses were confirmed via the contrast enhanced multi-detector computed tomography (MDCT) or magnetic resonance imaging (MRI), which were acquired prior to their TACE procedure. These were divided into two groups, the control group that consisted of DSA guidance alone; and the study group which consisted of DSA and CBCT imaging guidance (Figure 1). All TACE procedures were completed by four fellowship trained consultant radiologists (A, B, C and D) with TACE experience of 12, 10, 10 and 15 years, respectively. Within each procedure chemotherapeutic drugs were delivered via drug eluting beads (DEB) mixed with contrast medium, Omnipaque 300 (GE Healthcare; Chicago, USA).

**Table 1:** Inclusion and exclusion criteria.

Inclusion criteria:
Previous contrast enhanced MDCT or MRI, confirming HCC (Li-RADS 3 and above^(7)^).
TACE procedures that have been completed.
TACE procedures completed with a 6-s CBCT protocol.
TACE procedures completed without CBCT imaging.
Exclusion criteria:
TACE procedures for other diagnosis (e.g. cholangiocarcinoma and metastatic disease).
Incomplete and/or aborted TACE procedures.
TACE procedures completed with other CBCT protocols (including Dyna-PBV Body trial version).
TACE procedures with an additional completion CBCT imaging post procedure.

MDCT = multi-detector computed tomography, MRI = magnetic resonance imaging, HCC = hepatocellular carcinoma, TACE = trans-arterial chemoembolisation, CBCT = cone-beam computed tomography.

The systems flat panel detector was 30 × 40 cm and all CBCT imaging was acquired with the 6-s CBCT/DynaCT protocol. To ensure accuracy of dose readings from the angiographic equipment, a robust maintenance and quality assurance programme was in place that ensures the equipment is operating to relevant standards and manufacture specification. Quality assurance tests conducted by the medical physicist during the study period indicated that the dose area product (DAP) remained well within the 10% local tolerance limit at 0.7%.

Demographic data were collected through patient records and dose data from the RDSR.

### Statistical analysis

Once all data were retrieved, they were exported into the IBM SPSS statistical software (IBM SPSS Inc., Armonk, New York, USA) for analysis. Each variable was then tested for normal distribution and the appropriate statistical tests were performed. All data were then explored and tested for normal distribution using the Shapiro–Wilk test of normality. This test was selected because it is more appropriate for small sample sizes, as collected within this study. All parametric or normally distributed data were described with mean values and standard deviation; non-parametric data (data which were not normally distributed) were described with median values and interquartile ranges to better represent the data collected. As the majority of the dose data within this study were not parametric, the Mann–Whitney U test for comparison of two groups; and the Kruskal–Wallis test for comparisons across more than two groups, were chosen, with the exception being the Fischer’s Exact test that was used for binary data. Post-hoc Bonferroni correction for multiple comparisons was used to explore inter-operator fluoroscopy times. A *p*-value of ≤0.05 was deemed to be statistically significant.

## RESULTS

### Procedure enrolment

A total of 215 completed TACE procedures were identified for the treatment of HCC. 82 of these procedures fell within the control group; however, two of these procedures did not have the patient’s body weight recorded and another two procedures had missing RDSRs. Therefore, these four patients were excluded from the study, which led to a final number of 78 procedures within the control group.

The remaining 133 procedures fell into the study group, which was comprised of TACE procedures with both CBCT and DSA imaging guidance. However, when assessed alongside the eligibility criteria, 77 of these procedures had been performed with the trial CBCT protocol (a temporarily available trial version of Siemens Syngo Dyna Parenchymal Blood Volume Body (Dyna-PBV Body)), eight procedures were identified to have their CBCT repeated due to technical errors, three procedures did not have patients’ weight recorded, and one patient was without a RDSR. These 89 procedures were excluded from the study. A final number of 44 procedures were included within the study group (Figure 1).

### Patient and procedure characteristics

A total of 122 procedures were included within the study. 108 patients were male, and 14 patients were female. No statistically significant difference was found for the weight distribution for both groups (*p = 0.660*), and this allowed for direct comparison of exposure dose parameters between the two groups. Patient and procedure characteristics are summarised in Table 2.

**Table 2:** Baseline characteristics of patients (control group vs study group).

Characteristics	Control group (DSA guidance alone)	Study group (CBCT & DSA guidance)	*p* value
Age (years) Median Range	65 35–84	68 45–82	0.218
Gender Male (%) Female (%)	70 (89.7%) 8 (10.3%)	38 (86.4%) 6 (13.6%)	0.806
Weight (kg) Mean (SD) Range	86.8 (14.5) 55.8 (60.2–116.0)	86.5 (18.8) 76.3 (48.3–124.6)	0.660
HCC lesion quantity Median (IQR) Range	2 (1–3) 9 (1–10)	2.5 (0.5–4.5) 6 (1–7)	0.751
TACE First procedure (%) Subsequent procedure (%)	26 (33.3%) 52 (66.7%)	15 (34.1%) 29 (65.9%)	0.543

CBCT = cone-beam computed tomography; DSA = digital subtraction angiography; HCC = hepatocellular carcinoma; IQR = interquartile range; SD = standard deviation.

There was a statistically significant reduction in the number of DSA runs (3 vs 5, *p <* 0.001, Table 3) when comparing the study group to the control group. DSA PKA was also statistically significantly lower for the study group (3077.3 vs 4276.6 μGym^2^, *p = 0.042*; a 28% reduction) and corresponding lower DSA K_a,r_ (136.1 vs 153.3 mGy; an 11.2% reduction, Table 3). However, the DSA K_a,r_ difference was not found to be statistically significant.

**Table 3:** Median radiation dose comparison of TACE procedures with and without CBCT guidance.

Exposure variables	Image guidance		*p* value
	Control group (DSA only)	Study group (CBCT & DSA)	
Number of DSA runs Median (IQR)	5 (3–6)	3 (2–4)	0.001
DSA PKA (μGym^2^) Median (IQR)	4276.6 (2520.2–7218.8)	3077.3 (1076.3–6219.9)	0.042
DSA K_a,r_ (mGy) Median (IQR)	153.3 (90.1–298.2)	136.1 (36.2–246.8)	0.062
Procedure PKA (μGym^2^) Median (IQR)	6842.2 (4579.8–11931.5)	13778.0 (10255.8–18416)	0.001
Procedure K_a,r_ (mGy) Median (IQR)	322.7 (198.7–617.5)	557.8 (413.4–882.0)	0.001
Fluoroscopy time (min) Median (IQR)	14.2 (10.8–20.2)	13.4 (10.1–19.3)	0.833
Fluoroscopy PKA (μGym^2^) Median (IQR)	2297.8 (1354.0–4523.9)	2829.7 (2010.3–4133.7)	0.228
Fluoroscopy K_a,r_ (mGy) Median (IQR)	156.2 (82.2–361.9)	185.1 (133.5–275.9)	0.303
CBCT PKA (μGym^2^) Mean (SD) Range		7231.9 (1361.5) 6663 (3025.8–9688.8)	
CBCT K_a,r_ (mGy) Mean (SD) Range		232.9 (44.0) 214.7 (97.3–312.0)	

CBCT = cone-beam computed tomography; DSA = digital subtraction angiography; PKA = air kerma-area product; K_a,r_ = reference air kerma; SD = standard deviation.

Total procedural PKA (13778.0 vs 6842.2 μGym^2^, Table 3) and total procedural K_a,r_ (557.8 vs 322.7 mGy, Table 3) were shown to be, respectively, 50 and 73% higher within the study group (*p <* 0.001, Table 3). The average CBCT exposure dose alone accounted for more than 52% of total procedure PKA, as listed in Table 3. The fluoroscopy time differences were not found to be significantly different (*p =* 0.833, Table 3); neither were the fluoroscopy KAP (*p = 0.228*) and fluoroscopy K_a,r_ (*p = 0.303*, Table 3) between both groups.

For both the control and the study groups, the number of procedures performed per patient, and number of HCC lesions demonstrated no statistically significant relationship when compared with fluoroscopy time, total procedural PKA and total procedural K_a,r_ (Table 4). The effects of operator on fluoroscopy time were statistically significant (*p =* 0.003), with Bonferroni correction indicating significant differences in fluoroscopy times between operators D and A (*p =* 0.006), and operators B and A (*p =* 0.027). However, the total procedural PKA (*p* = 0.063) and K_a,r_ (*p* = 0.089) were not significantly affected by the operators, despite the difference in fluoroscopy times and procedure numbers across operator groups. Median fluoroscopy times for operators A to D were 16.9 (range: 11.9–25.3 min), 12.9 (range: 9.4–15.9 min), 14.2 (range: 12.2–19.1 min) and 11.1 min (range 9.2–17.7 min), respectively.

**Table 4:** Number of procedures and HCC lesions vs fluoroscopy time, total procedural PKA and K_a,r_.

	Fluoroscopy time (min)	Total procedural PKA (μGym^2^)	Total procedural K_a,r_ (mGy)
Procedure number (Mann–Whitney U test)	*p* = 0.759	*p* = 0.830	*p* = 0.564
Number of HCC lesions (Kruskal–Wallis test)	*p* = 0.371	*p* = 0.199	*p* = 0.098

HCC = hepatocellular carcinoma; PKA = air kerma-area product; min = minutes; K_a,r_ = reference air kerma.

## DISCUSSION

### DSA runs, air kerma-area product and reference air kerma analysis

The difference in the number of DSA runs was studied in view of previous investigations by Pitton *et al*.^(8)^ and Bartal *et al*.^(9)^, which demonstrated ~70% of total procedural PKA is contributed by DSA acquisitions. Our study demonstrated 62.5% of total procedural PKA and 47.5% of total procedural K_a,r_ is from DSA acquisition alone. The number of DSA runs and DSA PKA were shown to be significantly lower in the study group when compared to the control group. The non-significant lower DSA K_a,r_ data for the study group may be due to modifications in DSA acquisitions when CBCT imaging is used during TACE procedures. As CBCT imaging provides an overview, subsequent DSA acquisitions may be more super-selective in nature and can require magnified projections, leading to an increase in the K_a,r_.

Only one other study, to our knowledge, has examined the effects of CBCT imaging guidance in TACE procedures on the number of DSA runs, DSA PKA and DSA K_a,r_. The study was published by Kothary *et al*.^(10)^ and the results indicated a statistically significant reduction for all three variables described above (*p* < 0.05). The reduction in the number of DSA runs reported was from 5.4 runs in the control group to 4.4 runs in the study group (*p =* 0.007). The DSA PKA and DSA K_a,r_ readings from the Kothary *et al*.^(10)^ study were also found to be much higher when compared to the reported values in this study (3.1 and 4.9 times higher, respectively). The large difference in values between both studies were not expected as the values reported by the Kothary *et al*.^(10)^ study were normalised to patient body mass index (BMI) and, therefore, should be lower than values from the current study which were not normalised^(11)^; however, a key difference between the current study and that of Kothary et al. relates to the systems used with a newer generation of Siemens Artis system used in the current study. Possible factors contributing to the variability among study results are the difference in imaging requirements such as length of acquisition, collimation and acquisition frame rates; as well as equipment protocol and exposure settings chosen. There is also the issue of patient thickness or size when comparing dose readings between publications. BMI was used to normalise the exposure readings in the study by Kothary *et al*.^(10)^, whereas the current study used patient’s weight alone. Because of the statistical significance in the reduction in the number of DSA runs, the first null hypothesis is rejected in favour of the alternative hypothesis that CBCT imaging does reduce the number of DSA runs and DSA PKA in TACE procedures.

### Total procedural air kerma-area product and reference air kerma analysis

Statistically significant differences were found for the total procedural PKA and K_a,r_ (both *p <* 0.001). When comparing the exposure readings for both the study and the control groups, the PKA for the study group was double that of the control group (13778.0 vs 6842.2 μGym^2^) and the K_a,r_ for the study group was 73% higher than for the control group (557.8 vs 322.7 mGy). The dose from CBCT imaging alone was found to average at 52% (7231.9 μGym^2^) of the total procedural PKA and 42% of the total procedural K_a,r_ (232.9 mGy) respectively. Conversely, the difference in fluoroscopy times for both groups were not statistically significant (*p = 0.833*, Table 3).

K_a,r_ is a dose metric from which an indirect estimate of peak skin dose can be determined. An important point to note for the K_a,r_ comparison is that the dose to the patients’ skin from CBCT imaging is spread over a wider skin area due to the C-arm rotating around the patient during acquisition. This results in lower peak skin dose when compared to static DSA acquisitions. Unfortunately, comparison of reference doses from these two imaging techniques is challenging as there is limited availability of systems that accurately calculate the skin dose distribution. Therefore, caution should be applied when using the K_a,r_ data to infer differences in peak skin dose between CBCT and DSA runs.

Another possible use of CBCT imaging is the 3D image guidance overlay that is available within the system. After preliminary discussions with the operators, it was determined that its use may be suboptimal due to the large movement of the liver while the patient breathes. Albeit higher exposure doses were recorded within the study group, these doses were still within the diagnostic reference level (DRL) adopted by the department. As no national DRL was available for this procedure, this DRL was derived from a publication by Vano *et al*.^(12)^ on the European survey of radiation doses for interventional radiology and were also compared to those of the EUCLID (European Study on Clinical Diagnostic Reference Levels for X-ray Medical Imaging) study^(13)^ DRLs for hepatic embolisation/TACE; however, the EUCLID study does not differentiate between procedures with or without CBCT guidance. The doses in the current study for the control group (DSA only) and the study group (CBCT and DSA), as shown in Table 3, were all below the EUCLID DRLs (PKA = 24100 μGym^2^, K_a,r_ = 1868 mGy, fluoroscopy time = 18 mins). Bartal *et al*.^(9)^ have also recommended the use of CBCT imaging as a technique to reduce the number of DSA acquisitions and skin exposure dose concentrations.

### Operator, number of procedures and HCC lesion quantity analysis

There were significant differences in fluoroscopy time between operators, but the differences were not statistically significant for total procedural PKA and K_a,r_. It should be kept in mind that fluoroscopy time is a poor indicator of patient radiation exposure and should not be used as a form of dose monitoring unless no other exposure matrix is available. Treatment interval and HCC lesion quantity were also not significant when tested against fluoroscopy times, total procedural PKA and K_a,r_. The assumption that a secondary or repeat TACE procedure, with the help of previous imaging, would be easier to perform and would require less imaging was thus not proven. Possible explanations for this could be the alteration of tumour feeding vessels after TACE treatment, leading to an increase in difficulty for the next TACE procedure, and the limited sample sizes.

### Limitations

A limitation in this study was the use of weight alone, rather than BMI, as patient height was unavailable at the time of the study. Separately, peak skin dose calculations were not made in this study and as only K_a,r_ data were presented and current methods of estimating peak skin dose from K_a,r_ come with significant uncertainties. Thus, any inference in relation to skin dose would be inaccurate due to the differences in techniques and in relation to how the dose is distributed on the patients’ skin in CBCT vs DSA. Finally, the sample sizes, with 44 procedures in the study group is another limiting factor.

## CONCLUSION

The study has demonstrated that CBCT imaging guidance does reduce the number of DSA runs and DSA PKA required to complete the TACE procedure. However, a substantial increase in total procedural PKA is to be expected with the addition of CBCT imaging. The increase of total procedural K_a,r_ should also be analysed carefully as the K_a,r_ accumulated from CBCT imaging will lead to lower peak skin exposure by the very method that CBCT is acquired. Number of procedures and lesion quantity do not seem to affect procedural fluoroscopy times, total PKA nor K_a,r_. While recognising the many benefits of using CBCT, its inclusion in study protocols can increase the dose (both PKA and K_a,r_) to the patient significantly and it is thus important that this increased dose is carefully considered, justified, and protocols are optimised to ensure total procedural doses are kept as low as reasonably achievable (ALARA).
